# Oro-facial filariasis–A systematic review of the literature

**DOI:** 10.1371/journal.pntd.0012610

**Published:** 2024-11-06

**Authors:** Agnesa Bytyqi, Chiara Karas, Klara Pechmann, Michael Ramharter, Johannes Mischlinger

**Affiliations:** 1 Center for Tropical Medicine, Bernhard Nocht Institute for Tropical Medicine & I. Department of Medicine University Medical Center Hamburg-Eppendorf, Hamburg, Germany; 2 German Centre for Infection Research, Partner Site Hamburg-Lübeck-Borstel-Riems, Hamburg-Lübeck-Borstel-Riems, Hamburg, Germany; 3 Centre de Recherches Médicales de Lambaréné, Lambaréné, Gabon; Colorado State University, UNITED STATES OF AMERICA

## Abstract

**Introduction:**

Filarial pathogens are described to inhabit and affect subcutaneous and lymphatic tissues of the human host. To date, little is known on how much oral health might be affected by filarial infections, even though involvement of the oro-facial region is pathophysiologically possible. Therefore, we conducted this systematic review of the literature to help reduce the current evidence gap. First, we reviewed the existing literature related to oro-facial filariasis and summarized all confirmed cases in detail. Second, we presented the demographic clinical characteristics of published oro-facial filariasis cases using descriptive statistics.

**Methods:**

A comprehensive search was conducted using PubMed and Google Scholar to identify scholarly articles on oro-facial filariasis (PROSPERO: CRD42024551237). Clinical trial registries of clinicaltrials.gov and the Pan-African Clinical Trials Registry (PACTR) were checked for ongoing studies on oro-facial filariasis.

From clinical articles on filariasis and oro-facial health, patient-specific information was ascertained such as country of diagnosis, age, sex and symptoms of the patient, location of filarial disease manifestation, filarial worm species diagnosis, main clinical diagnosis, as well as main pathology and lastly therapy. Descriptive statistics were computed.

**Results:**

The systematic search was conducted on 18.06.2024. Initially a total of 1,064 publications was identified. No registered study on oro-facial filariasis was found on large clinical trial registers. After sequentially assessing abstracts and full-texts for eligibility, the analysis population was reduced to 68 articles amounting to 111 cases of oro-facial filariasis. Published articles which were identified and ultimately selected consisted solely of case reports, or case series; not a single epidemiological study was found in the published body of literature. Published data on oro-facial filariasis was identified from as early as 1864 until 2022. The median age of oro-facial filariasis cases was 39 years (range: 1 year to 80 years) and evenly distributed between the two sexes (49% [54/110] female and 51% [56/110]; sex not reported for one case). The vast majority of identified cases was on oro-facial dirofilariasis (92% [102/111]), followed by lymphatic filariasis (2.5% [3/111]), lymphatic filariasis with squamous carcinoma (2.5% [3/111]), and lastly by onchocerciasis (1% [1/111]). Although in 34% (38/111) of articles there was no clear description of the main pathology of oro-facial filariasis, all of the remaining 73 articles described nodules or swellings. Asymptomatic manifestations constituted almost 75% (55/73) and only about 25% (18/73) of articles described a symptomatic case.

**Conclusion:**

Although filarial diseases are to date not generally regarded as being associated with oral health problems this assumption might not be justified. This comprehensive systematic review was conducted to detect and collate all published studies on oro-facial filariasis. The fact that only case reports, or case series were identified suggests that this constitutes a neglected field of research. Cases identified in the published literature indicate that the vast majority of published oro-facial, filarial case reports were cases of dirofilariasis. Among the published studies, oro-facial filariasis manifested exclusively as nodules or swellings in different tissue locations. These nodules and swellings were mostly asymptomatic and therefore, cancer is an important differential diagnosis.

## Author summary

Although, filarial parasites can in theory affect the oro-facial space little is to date known on whether oro-facial involvement occurs in the course of filarial infection. Therefore, we conducted a review of the published scientific literature to provide a first estimate on the characteristics of oro-facial filariasis. We identified 68 articles published between 1864 and 2022 totaling up to 111 cases, which we further summarized and described in detail. The fact that the article type ‘case report’ was the solely published study design suggests that this constitutes a neglected research topic. The vast majority of patients had oro-facial dirofilariasis which manifested exclusively as nodules or swellings in different tissue locations. These nodules and swellings were mostly asymptomatic and therefore, cancer is an important differential diagnosis. Further prospective studies are now needed to understand better the frequency, nature, and public health impact of oro-facial filariasis.

## Introduction

### Rationale

Approximately 170 million people worldwide suffer from filariasis [1]. Various nematode species within the filarioidea superfamily are responsible for these infections, which are almost exclusively transmitted to humans through the bite of an insect vector. Adult filariae typically reside in the subcutaneous and lymphatic tissue of human hosts, where they produce juvenile stages, so called microfilariae through sexual reproduction. Depending on the species, microfilariae can be found in the blood or subcutaneous tissue. The presence of microfilariae in the small blood vessels of the skin, or in subcutaneous tissue allows them to infect different insect vectors during a blood meal. Humans can be infected by several species of filariae, however, *Wuchereria bancrofti*, *Brugia malayi*, *Onchocerca volvulus* and *Loa loa* are responsible for the majority of severe filarial infections [1]. Besides, other filarial species can infect humans as well, but in most such cases humans act as accidental or dead-end hosts; this means that parasites cannot sexually multiply in such hosts, who thereby cannot pass on the infection to other vectors anymore [2].

Infection typically occurs when an individual is repeatedly exposed to infective larvae over an extended period. The clinical symptoms develop gradually, making filariasis a chronic infection with potential long-term consequences depending on the infective species and the affected organ system. Filariasis is most commonly an endemic disease that occurs particularly in lower- and middle-income countries of tropical and subtropical regions [3]. The diversity of genera results in various clinical manifestations in humans and oral involvement is believed to be very rare. Despite its widespread overall occurrence and significant disease burden, filariasis is classified as one of the 21 "neglected tropical diseases" by the World Health Organization (WHO) [4,5].

Although highly prevalent worldwide, oral diseases also constitute a neglected field of global health. According to the Global Oral Health Status Report 3.5 billion people suffered from oral diseases, most commonly dental caries, severe gum disease, tooth loss and oral cancers [6]. Several infectious agents are known to manifest in the oral cavity with unspecific or pathognomonic signs. Viral, bacterial, fungal and protozoan pathogens have been described to cause or manifest in the oral cavity [7]. Cysticercosis and trichinosis constitute two exemplary helminthic diseases with potential involvement of the oral region; the former is caused by the cestode *Taenia solium* and the latter by the nematode *Trichinella spiralis* [8,9]. However, to date little is known about filarial pathogens and their impact on oral, or oro-facial health, even though pathophysiologically it is plausible that adult or juvenile filarial stages might cause oro-facial health problems when being anatomically located in the head region.

This shall be demonstrated by looking at subcutaneous dirofilariasis, a filarial disease caused by *Dirofilaria species* (*D*. *repens* and *D*. *tenuis*) for whom domestic dogs and wild canids are the definitive host. During a blood meal taken from an infected canid, infective larvae are ingested by mosquito species (e.g. *Aedes spp*., *Anopheles spp*., or *Mansonia spp*.) which ultimately renders them infectious [2,10]. During a subsequent bloodmeal taken from humans, infective dirofilarial larvae are released into the human accidental host in whom larvae eventually develop into adults which migrate in the subcutaneous tissue, or form granulomatous nodules there. If manifesting in the oro-facial region subcutaneous dirofilariasis could be etiologically important in explaining oro-dental clinical phenomena, such as swellings, nodules and potentially even (tooth) pain, caused by edematous swelling exerted onto local nerves by a migrating adult worm, or a juxtaposed granulomatous nodule. Local pressure might also inhibit blood flow, which in theory could cause dental ischemia and thereby possibly lead to tooth loss. Lastly, it is theoretically possible that systemic or local inflammatory activity caused by filarial infection creates a procancerogenic environment potentially contributing to the formation of oral cancer.

To the best of our knowledge no systematic research effort has yet been conducted in this field. Therefore, we performed this systematic review of the literature to help reduce the current evidence gap. The first objective was to review the existing literature related to oro-facial filariasis and summarize all confirmed cases in detail. The second objective was to present the demographic clinical characteristics of published oro-facial filariasis cases using descriptive statistics.

## Methods

### Eligibility criteria

An article was included if it was a human filarial infection in the oro-facial region. Concordantly, any filarial infection was excluded which does not affect the face or mouth, or is a non-human infection.

### Information sources

A comprehensive search was conducted using the PubMed database and Google Scholar to identify scholarly articles on oro-facial filariasis. Articles were included if focusing on human filariasis and oro-facial health. Articles of all languages were eligible for inclusion. A review protocol of this project was registered on PROSPERO (CRD42024551237).

Clinical trial registries of clinicaltrials.gov and the Pan-African Clinical Trials Registry (PACTR) were checked for ongoing studies using the key words oral filariasis, oral filarial disease, oro-facial filariasis, oro-facial filarial disease, oral loiasis, oral mansonellosis, oral elephantiasis, oral onchocerciasis, oral dirofilariasis.

### Search strategy

A systematic search included following key words of two categories: A) Filariasis: *Filarioidea*, *Brugia*, *Brugia malayi*, *Dirofilaria*, *Dirofilaria immitis*, *Dirofilaria repens*, *Dirofilaria tenuis*, *Loa*, *Loa loa*, loiasis, microfilaria, *Onchocerca*, *Onchocerca volvulus*, onchocerciasis, *Mansonella*, mansonellosis, *Mansonella ozzardi*, *Mansonella perstans*, *Mansonella streptocerca*, *Wuchereria*, *Wuchereria bancrofti*, filariasis, filariosis, filarial disease, elephantiasis. B) Oro-facial health: dental, dental problems, mouth, teeth, oral, maxilla, mandibula.

These keywords were combined with the Boolean operators "AND" and "OR" and MeSH terms were used in the PubMed search query. The search query on PubMed was as follows: ((filarioidea[MeSH Terms]) OR (brugia[MeSH Terms]) OR (brugia malayi[MeSH Terms]) OR (dirofilaria[MeSH Terms]) OR (dirofilaria immitis[MeSH Terms]) OR (dirofilaria repens[MeSH Terms]) OR (loa[MeSH Terms]) OR (mansonella[MeSH Terms]) OR (microfilariae[MeSH Terms]) OR (onchocerca[MeSH Terms]) OR (onchocerca volvulus[MeSH Terms]) OR (wuchereria[MeSH Terms]) OR (wuchereria bancrofti[MeSH Terms]) OR (filariasis[MeSH Terms]) OR (dirofilariasis[MeSH Terms]) OR (elephantiasis, filarial[MeSH Terms]) OR (loiasis[MeSH Terms]) OR (mansonelliasis[MeSH Terms]) OR (onchocerciasis[MeSH Terms]) OR (filarioidea) OR (brugia) OR (brugia malayi) OR (brugia timori) OR (Dirofilaria) OR (dirofilaria immitis) OR (dirofilaria repens) OR (dirofilaria tenuis) OR (loa) OR (loa loa) OR (mansonella ozzardi) OR (mansonella perstans) OR (mansonella streptocerca) OR (microfilariae) OR (onchocerca) OR (onchocerca volvulus) OR (wuchereria) OR (wuchereria bancrofti) OR (filariasis) OR (filarial disease) OR (dirofilariasis) OR (elephantiasis, filarial) OR (loiasis) OR (mansonelliasis) OR (onchocerciasis)) AND ((mouth[MeSH Terms]) OR (tooth[MeSH Terms]) OR (maxilla[MeSH Terms]) OR (mandible[MeSH Terms]) OR (jaw[MeSH Terms]) OR (dental) OR (dental problems) OR (mouth) OR (tooth) OR (maxilla) OR (mandible) OR (articulatio temporomandibularis) OR (temporomandibular joint) OR (oral cavity)).

Initially, an attempt was made to use the PubMed search query in the same way on Google Scholar. However, due to an enormous lack of specificity of the PubMed search query when used on Google Scholar it needed to be modified as follows: (Filarioidea) OR (Brugia) OR (Brugia malayi) OR (Brugia timori) OR (Dirofilaria) OR (Dirofilaria immitis) OR (Dirofilaria repens) OR (Dirofilaria tenuis) OR (Loa) OR (Mansonella ozzardi) OR (Mansonella perstans) OR (Mansonella streptocerca) OR (Onchocerca) OR (Onchocerca volvulus) OR (Wuchereria) OR (Wuchereria bancrofti) OR (Filariasis) OR (filarial disease) OR (Dirofilariasis) OR (Elephantiasis) OR (Loiasis) OR (Mansonelliasis) OR (Onchocerciasis)) AND (dental) OR (dental problems) OR (mouth) OR (maxilla) OR (mandible) OR (jaw).

### Study records

All articles detected by the search strategy were subjected to title and abstract screening by two researchers. If eligibility criteria were violated the article was excluded from the analysis population. In case of discordances in article eligibility between first and second researcher, a third researcher decided on final article selection. Subsequently, the full texts of remaining articles were read again by two-to-three independent researchers and were excluded from the analysis population if the eligibility criteria were violated. In addition, the references of the articles remaining in the analysis population were manually searched for potentially eligible manuscripts and included if eligible. In case of non-English articles, ‘Google Translate’ (Google LLC, California, USA) was used to translate the article into English.

### Data items and outcomes

From clinical articles on filariasis and oro-facial health, patient-specific information was ascertained such as country of diagnosis, age, sex and symptoms of the patient, location of filarial disease manifestation, filarial worm species diagnosis, main clinical diagnosis, as well as main pathology and lastly therapy. Descriptive statistics were performed with MS Excel (Redmond, Washington, USA) and STATA17 (StataCorp, Texas, USA).

### Risk of bias assessment

A preliminary search indicated that we would primarily detect case reports and case series. Therefore, we used a tool developed by Murad et al. which was developed for systematic reviews collecting evidence primarily from case reports and case series (8). It includes evaluation of eight items from four domains: selection, ascertainment, causality and reporting. Each item is based on a leading question and scores with either 0 or 1, and scores were summed up into an aggregate score (range 0–8 points). Risk of bias was assessed by two independent reviewers. Based on the results of the evaluated eight items, each reviewer made an overall statement of the methodological quality of each assessed report. This statement was one out of three categories (low, acceptable, high) and did not have to be based on the sum of the aggregated score, as not each item was of equal importance for this systematic review (e.g. questions 5–7 are designed for case reports of adverse drug side effects). Therefore, the overall methodological categorization was based on a qualitative assessment. Potential discrepancies in methodological categorization between first and second reviewer were resolved by a third reviewer.

## Results

**Fig 1:**
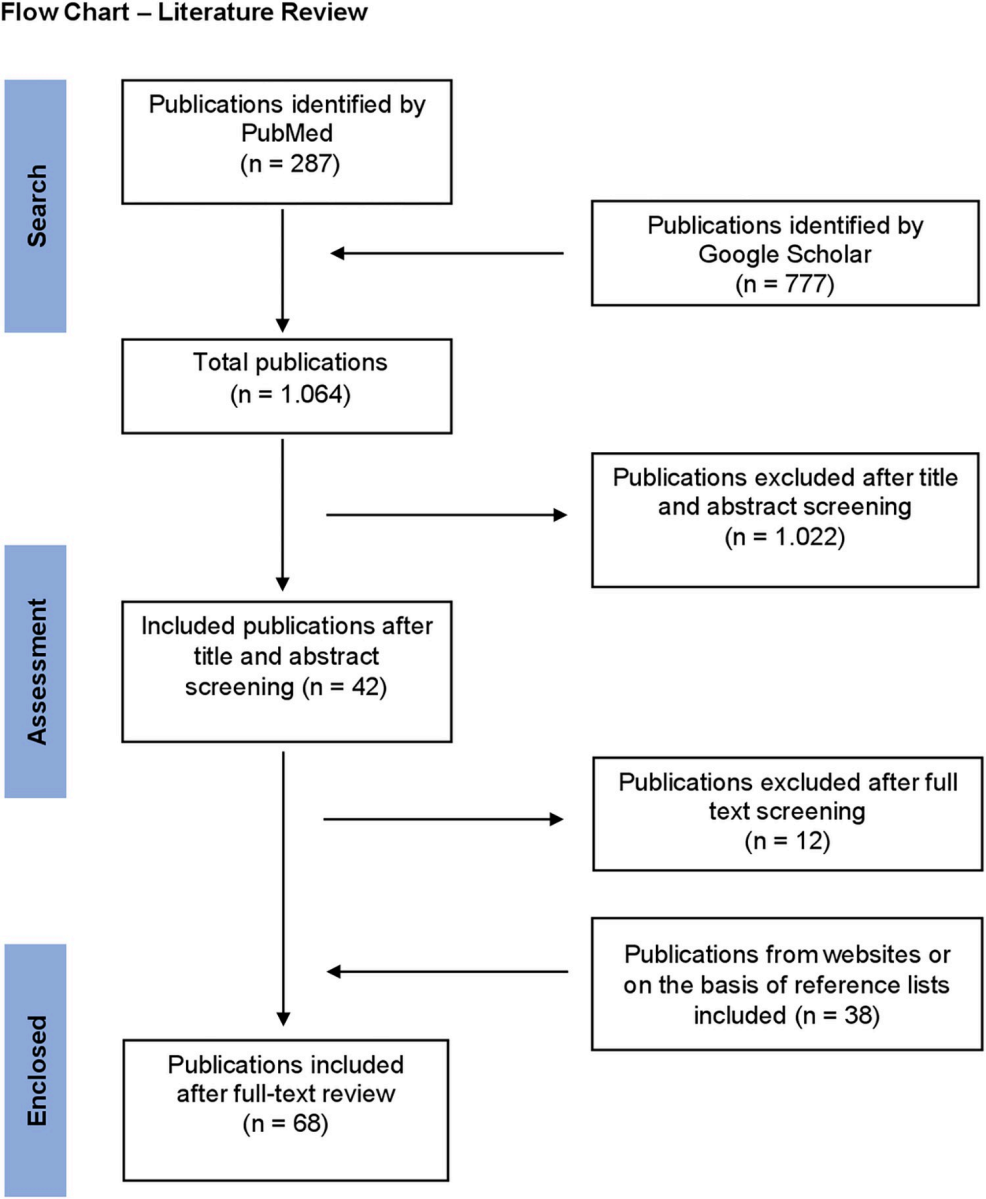
Flow Chart–Literature Review.

The systematic search was conducted on 18.06.2024. Searches on PubMed and Google Scholar identified a total of 1,064 publications (Fig 1). PubMed identified 287 results and Google Scholar 777. After assessing titles and abstracts of these articles for eligibility the analysis population was reduced to 42 articles. Subsequently, the full texts of these 42 manuscripts were reviewed and further twelve articles were excluded. Finally, 38 articles were added by being identified through the reference list of respective full texts. Data was then extracted and compiled from a total of 68 publications amounting to 111 cases of oro-facial filariasis.

No registered study on oro-facial filariasis was found on clinicaltrials.gov or the Pan-African Clinical Trials Registry (PACTR).

As anticipated from a preliminary search, we only detected published articles on case reports, case series or review articles which had already partially compiled existing case reports. Published data on oro-facial filariasis was identified from as early as 1864 until 2022 (Tables 1 and 2). It seems that oro-facial filariasis can affect people of any age, as indicated by a median 39 years with an age range of 1 year to 80 years. It appears that both sexes are equally often affected, as demonstrated by 49% (54/110) cases being female and 51% (56/110) being male; sex was not reported for one case. The vast majority of identified cases was on oro-facial dirofilariasis (92%, 102/111), followed by lymphatic filariasis (2.5%; (3/111)) and lymphatic filariasis with squamous carcinoma (2.5%; 3/111), followed by onchocerciasis (1%, 1/111). Two articles mentioned filariasis, however, did not further specify the disease or filarial pathogen (2%, 2/111).

**Table 1:** Descriptive statistics on all detected case reports.

Characteristic	Dirofilariasis (N = 102)	Filariasis (N = 2)	Lymphatic filariasis (N = 3)	Onchocerciasis (N = 1)	Squamous cell carcinoma and lymphatic filariasis (N = 3)
Year of case report publication (median; [range])	2003 (1864 to 2022)	2001 and 2017	2001, 2007 and 2010	1986	1985, 1985 and 1998
1864 to 1989	21 (20.6%)	0	0	1 (100%)	2 (67%)
1990 to 1999	26 (25.5%)	0	0	0	1 (33%)
2000 to 2009	22 (21.6%)	1 (50%)	2 (67%)	0	0
2010 to present	33 (32.3%)	1 (50%)	1 (33%)	0	0
Age in years (median; [range])	39 (1 to 80)	44 and 46	34, 35 and 50	13	45, 54 and 62
1 to 19	11 (11%)	0	0	1 (100%)	0
20 to 39	42 (41%)	0	2 (67%)	0	0
40 to 59	35 (35%)	2 (100%)	1 (33%)	0	2 (67%)
60 and older	13 (13%)	0	0	0	1 (33%)
N/A†	1	0	0	0	0
Sex (n [%])					
male	49 (49%)	1 (50%)	3 (100%)	1 (100%)	0
female	52 (51%)	1 (50%)	0	0	3 (100%)
N/A†	1	0	0	0	0
Region (according to WHO) (n [%])					
African Region	0	0	0	1 (100%)	2 (67%)
Eastern Mediterranean Region	3 (2.9%)	0	0	0	0
European Region	55 (53.9%)	0	0	0	0
Region of the Americas	5 (4.9%)	0	0	0	0
South-East Asian Region	38 (37.3%)	1 (50%)	3 (100%)	0	1 (33%)
Western Pacific Region	1 (1%)	1 (50%)	0	0	0
Filarial worm species diagnosis (n [%])					
*Dirofilaria repens*	81 (98%)	0	0	0	0
*Dirofilaria tenuis*	2 (2%)	0	0	0	0
*Onchocerca volvulus*	0	0	0	1 (100%)	0
*Wuchereria bancrofti*	0	0	3 (100%)	0	3 (100%)
N/A†	19	2	0	0	0
Location (n [%])					
Cheek	65 (63.7%)	1 (50%)	2 (67%)	1 (100%)	1 (33%)
Face	18 (17.7%)	0	0	0	0
Jaw	7 (6.9%)	1 (50%)	0	0	0
Lip	8 (7.8%)	0	0	0	1 (33%)
Other	4 (3.9%)*	0	1 (33%)**	0	1 (33%)***
Main pathology (n [%])					
Asymptomatic nodule	29 (45.3%)	1 (50%)	1 (33%)	1 (100%)	0
Asymptomatic swelling	21 (32.8%)	0	2 (67%)	0	0
Symptomatic nodule	4 (6.3%)	0	0	0	1 (33%)
Symptomatic swelling	10 (15.6%)	1 (50%)	0	0	2 (67%)
N/A†	38	0	0	0	0
Main symptom (for symptomatic cases) (n [%])					
Dysesthesia	1 (7.1%)	0	0	0	0
Pain	9 (64.3%)	1 (100%)	0	0	1 (33%)
Pruritus	4 (28.6%)	0	0	0	0
Ulcer	0	0	0	0	2 (67%)
Therapy (n [%])					
Fine needle biopsy	0	0	1 (33%)	0	1 (100%)
Surgical removal	49 (89%)	1 (50%)	1 (33%)	1 (100%)	0
Surgical removal and anti-filarial oral treatment	3 (5%)	1 (50%)	1 (33%)	0	0
Swelling was squeezed and the worm was recovered	2 (4%)	0	0	0	0
The nodule developed into an abscess, which burst and revealed a white worm	1 (2%)	0	0	0	0
N/A†	47	0	0	0	2

N/A = Not available

†The number of cases for whom data was not available was excluded from computation of relative percentages

*Locations were nasolabial region, oral cavity, soft palate, tongue

** Location was gingiva

*** Location was floor of the mouth

**Table 2:** Detailed case descriptions sorted by publication year, country and diagnosis.

Publication year	Country	Age	Sex	Location	Case description	Diagnosis	Species	Treatment	Methodological quality	Ref.
1864	Italy	20	M	Lip	n.a.	Dirofilariasis	*Dirofilaria repens*	n.a.	Low	[11]
1937	Italy	18	M	Cheek	n.a.	Dirofilariasis	*Dirofilaria repens*	n.a.	Low	[11]
1952	Greece	26	F	Cheek	n.a.	Dirofilariasis	n.a.	n.a.	Low	[12]
1966	Sri Lanka	48	M	Face	n.a.	Dirofilariasis	n.a.	n.a.	Low	[13]
1967	Italy	71	M	Cheek	n.a.	Dirofilariasis	*Dirofilaria repens*	n.a.	Low	[11]
1967	Italy	54	F	Cheek	n.a.	Dirofilariasis	*Dirofilaria repens*	n.a.	Low	[11]
1968	Italy	20	M	Face	n.a.	Dirofilariasis	*Dirofilaria repens*	n.a.	Low	[11]
1982	France	42	M	Cheek	Firm, insensitive lump on the right cheek	Dirofilariasis	*Dirofilaria repens*	Surgical removal	High	[14]
1982	France	35	F	Face	n.a.	Dirofilariasis	*Dirofilaria repens*	n.a.	Low	[11]
1983	France	19	M	Cheek	n.a.	Dirofilariasis	*Dirofilaria repens*	n.a.	Low	[11]
1983	Georgia	28	F	Tongue	n.a.	Dirofilariasis	*Dirofilaria repens*	n.a.	Low	[11]
1984	Russia	31	M	Cheek	Small submucosal tumor in the right cheek which has disappeared; after five months a thickened, itchy swelling appeared near the nasolabial fold and the face became asymmetrical	Dirofilariasis	*Dirofilaria repens*	Surgical removal	High	[15]
1985	Italy	40	M	Face	n.a.	Dirofilariasis	*Dirofilaria repens*	n.a.	Low	[11]
1985	Italy	44	M	Face	n.a.	Dirofilariasis	*Dirofilaria repens*	n.a.	Low	[11]
1986	France	50	M	Face	n.a.	Dirofilariasis	*Dirofilaria repens*	n.a.	Low	[11]
1986	Russia	55	F	Lip	Feeling of pain and tightness on the lower lip	Dirofilariasis	n.a.	Surgical removal	Acceptable	[16]
1987	France	47	M	Cheek	n.a.	Dirofilariasis	*Dirofilaria repens*	n.a.	Low	[11]
1987	Italy	25	M	Jaw	n.a.	Dirofilariasis	*Dirofilaria repens*	n.a.	Low	[11]
1987	Italy	23	M	Jaw	n.a.	Dirofilariasis	*Dirofilaria repens*	n.a.	Low	[17]
1988	Italy	37	F	Cheek	n.a.	Dirofilariasis	*Dirofilaria repens*	n.a.	Low	[11]
1989	Russia	18	F	Cheek	Swelling on the left cheek	Dirofilariasis	*Dirofilaria repens*	Surgical removal	High	[15]
1990	Greece	48	F	Cheek	Eight-month-old hard subcutaneous nodule on the left cheek	Dirofilariasis	*Dirofilaria repens*	Surgical removal	High	[18]
1991	France	38	F	Face	n.a.	Dirofilariasis	*Dirofilaria repens*	n.a.	Low	[11]
1991	Russia	41	F	Face	n.a.	Dirofilariasis	n.a.	n.a.	Low	[19]
1992	England	12	M	Face	Painful swelling in the region of the left zygomatic arch for four months	Dirofilariasis	n.a.	Surgical removal	Acceptable	[20]
1993	Florida (USA)	n.a.	n.a.	Cheek	Asymptomatic, two-month-old, firm swelling in the left maxilla	Dirofilariasis	*Dirofilaria tenuis*	Surgical removal	High	[21]
1993	France	39	M	Cheek	n.a.	Dirofilariasis	*Dirofilaria repens*	n.a.	Low	[11]
1993	France	56	M	Cheek	n.a.	Dirofilariasis	*Dirofilaria repens*	n.a.	Low	[11]
1993	Italy	54	M	Lip	Asymptomatic nodule with changing location: cheek, face, head, upper lip	Dirofilariasis	*Dirofilaria repens*	Surgical removal	High	[22]
1993	Russia	35	F	Soft palate	Increasing, painful and itching swelling on the soft palate	Dirofilariasis	*Dirofilaria repens*	Surgical removal	High	[15]
1993	Sri Lanka	28	M	Cheek	n.a.	Dirofilariasis	n.a.	n.a.	Low	[23]
1994	Italy	63	F	Cheek	n.a.	Dirofilariasis	*Dirofilaria repens*	n.a.	Low	[11]
1994	Italy	38	M	Face	n.a.	Dirofilariasis	*Dirofilaria repens*	n.a.	Low	[11]
1994	Sri Lanka	3	M	Cheek	n.a.	Dirofilariasis	n.a.	n.a.	Low	[23]
1995	Florida (USA)	66	M	Cheek	Asymptomatic mass in the cheek	Dirofilariasis	*Dirofilaria tenuis*	n.a.	Acceptable	[24]
1996	Italy	38	M	Face	n.a.	Dirofilariasis	*Dirofilaria repens*	n.a.	Low	[17]
1996	Russia	35	M	Jaw	Tumor in the submandibular region	Dirofilariasis	n.a.	Surgical removal	Acceptable	[16]
1996	Sri Lanka	1	F	Cheek	n.a.	Dirofilariasis	n.a.	n.a.	Low	[23]
1998	Italy	59	M	Jaw	n.a.	Dirofilariasis	*Dirofilaria repens*	n.a.	Low	[25]
1999	France	66	M	Cheek	n.a.	Dirofilariasis	*Dirofilaria repens*	n.a.	Low	[17]
1999	India	39	F	Lip	Painful swelling below the right angle of the lip; headache, urticaria and ear discharge since six months	Dirofilariasis	*Dirofilaria repens*	Swelling was squeezed and the worm was recoverd	High	[26]
1999	Italy	39	M	Face	n.a.	Dirofilariasis	*Dirofilaria repens*	n.a.	Low	[17]
1999	Italy	27	F	Cheek	n.a.	Dirofilariasis	*Dirofilaria repens*	n.a.	Low	[17]
1999	Italy	4	M	Cheek	n.a.	Dirofilariasis	*Dirofilaria repens*	n.a.	Low	[17]
1999	Sri Lanka	65	F	Cheek	n.a.	Dirofilariasis	*Dirofilaria repens*	n.a.	Low	[27]
1999	Sri Lanka	45	M	Cheek	n.a.	Dirofilariasis	*Dirofilaria repens*	n.a.	Low	[27]
1999	Sri Lanka	80	F	Cheek	Diffuse swelling involving the mucosa of the left face for one month increasing in size gradually	Dirofilariasis	*Dirofilaria repens*	Surgical removal	High	[28]
2000	Austria	59	M	Cheek	n.a.	Dirofilariasis	*Dirofilaria repens*	n.a.	Low	[29]
2002	Turkey	62	M	Jaw	Painful swelling for two months of the right cheek, lateral to the masseter	Dirofilariasis	*Dirofilaria repens*	Surgical removal	High	[30]
2003	China	42	F	Cheek	Six months of submucosal, firm, mobile swelling in the right maxilla	Dirofilariasis	*Dirofilaria repens*	Surgical removal	High	[31]
2003	Sri Lanka	26	F	Cheek	Lesion of the buccal mucosa	Dirofilariasis	*Dirofilaria repens*	Surgical removal	High	[32]
2003	Sri Lanka	80	F	Cheek	Swelling of buccal mucosa	Dirofilariasis	*Dirofilaria repens*	Surgical removal	High	[32]
2003	Sri Lanka	52	F	Cheek	Lump hard in consistency on buccal mucosa for five months	Dirofilariasis	*Dirofilaria repens*	Surgical removal	High	[32]
2003	Sri Lanka	28	F	Cheek	Intraoral nodule in the buccal mucosa	Dirofilariasis	*Dirofilaria repens*	Surgical removal	High	[32]
2003	Sri Lanka	4	F	Cheek	Submucosal, growing, intraoral nodule on the buccal mucosa	Dirofilariasis	*Dirofilaria repens*	Surgical removal	High	[32]
2003	Sri Lanka	40	F	Cheek	Intraoral, slow enlarging, asymptomatic lump on the buccal mucosa	Dirofilariasis	*Dirofilaria repens*	Surgical removal	High	[32]
2003	Sri Lanka	52	M	Lip	Fixed nodule in the lower lip	Dirofilariasis	*Dirofilaria repens*	Surgical removal	High	[32]
2004	Florida (USA)	73	F	Cheek	Right-sided facial mass since one month	Dirofilariasis	*Dirofilaria repens*	Surgical removal	High	[33]
2004	Russia	23	F	Oral cavity	n.a.	Dirofilariasis	*Dirofilaria repens*	n.a.	Low	[34]
2004	Russia	43	F	Cheek	n.a.	Dirofilariasis	*Dirofilaria repens*	n.a.	Low	[34]
2005	India	35	M	Lip	Painful facial swelling over three days, a thread-like structure appeared after the patient removed a blister on his lip	Dirofilariasis	*Dirofilaria repens*	Swelling was squeezed and the worm was recoverd	High	[35]
2006	Iran	34	M	Cheek	A single, firm and moveable nodule on the right cheek	Dirofilariasis	*Dirofilaria repens*	Surgical removal	High	[36]
2006	Iran	31	M	Nasolabial	Asymptomatic, solid tumour on the left side of the paranasal sinus	Dirofilariasis	*Dirofilaria repens*	Surgical removal	High	[37]
2006	Russia	32	F	Cheek	Subcutaneous nodule in the cheek	Dirofilariasis	*Dirofilaria repens*	n.a.	Acceptable	[38]
2007	France	35	M	Cheek	Subcutaneous nodule of the left cheek	Dirofilariasis	*Dirofilaria repens*	Surgical removal	High	[39]
2007	Russia	38	F	Face	Painless subcutaneous nodule in the zygomatic region	Dirofilariasis	*Dirofilaria repens*	n.a.	Acceptable	[38]
2008	Austria	62	F	Cheek	n.a.	Dirofilariasis	*Dirofilaria repens*	n.a.	Low	[29]
2009	Austria	52	M	Cheek	Two-month history of progressive swelling of the right cheek	Dirofilariasis	*Dirofilaria repens*	Surgical removal and prescription of albendazole	High	[40]
2009	Greece	46	F	Cheek	Painless subcutaneous hard and mobile nodule on the right cheek	Dirofilariasis	*Dirofilaria repens*	Surgical removal	High	[41]
2010	England	32	M	Face	Large swelling of the left face for several months	Dirofilariasis	n.a.	Surgical removal	Acceptable	[42]
2010	India	45	M	Cheek	Asymptomatic, tense and increasing swelling in the right cheek since one month	Dirofilariasis	*Dirofilaria repens*	Surgical removal	High	[43]
2010	Russia	30	F	Face	Painless subcutaneous nodule in the parotid region	Dirofilariasis	*Dirofilaria repens*	n.a.	Acceptable	[38]
2010	Tunisia	40	F	Lip	Subcutaneous lesion of the upper lip resembling a sebaceous cyst	Dirofilariasis	*Dirofilaria repens*	Surgical removal	High	[44]
2011	India	39	F	Face	Lymph node in the parotid region for two months	Dirofilariasis	*Dirofilaria repens*	n.a.	Acceptable	[45]
2011	India	10	F	Face	Cyst in the left nasolabial fold for two months	Dirofilariasis	*Dirofilaria repens*	n.a.	Acceptable	[45]
2011	India	45	F	Cheek	Lipoma in the cheek for one month	Dirofilariasis	*Dirofilaria repens*	n.a.	Acceptable	[45]
2011	India	40	M	Cheek	Nodule above the cheek since three months	Dirofilariasis	*Dirofilaria repens*	n.a.	Acceptable	[46]
2013	India	28	M	Cheek	Gradually increasing subcutaneous nodule on the left cheek for two weeks	Dirofilariasis	*Dirofilaria repens*	Surgical removal	High	[47]
2013	India	30	M	Cheek	Gradually increasing swelling for one year on the right cheek	Dirofilariasis	*Dirofilaria repens*	n.a.	Acceptable	[48]
2013	India	27	F	Cheek	Subcutaneous nodule on the right cheek for six months, discomfort in the neck and scalp with a crawling sensation under the skin for one year	Dirofilariasis	*Dirofilaria repens*	The nodule developed into an abscess, bursted and revealed a white worm	High	[49]
2013	India	54	F	Cheek	Eight months old, periodic, intraoral, itchy swelling on the left side	Dirofilariasis	*Dirofilaria repens*	Surgical removal and prescription of albendazole, diethylcarbamazine and cetrizine	High	[50]
2014	France	52	F	Jaw	Swelling of the left cheek for two weeks	Dirofilariasis	*Dirofilaria repens*	Surgical removal	High	[51]
2014	India	54	F	Cheek	Eleven months old, periodic, itchy swelling of the left cheek	Dirofilariasis	*Dirofilaria repens*	Surgical removal	High	[50]
2014	India	32	M	Cheek	Non tender diffuse swelling on the right cheek since one month	Dirofilariasis	n.a.	Surgical removal	Acceptable	[52]
2014	India	19	F	Cheek	Asymptomatic swelling of the left buccal mucosa for five months with spontaneous reduction and increase in swelling	Dirofilariasis	*Dirofilaria repens*	Surgical removal	High	[53]
2015	Brazil	65	F	Cheek	Submucosal painful nodule on the right buccal mucosa	Dirofilariasis	n.a.	Surgical removal	Acceptable	[54]
2015	India	32	M	Cheek	Asymptomatic, submucosal swelling of the right buccal mucosa	Dirofilariasis	*Dirofilaria repens*	Surgical removal	High	[55]
2015	India	32	M	Cheek	Persistent swelling in the lower right mandible region since one month	Dirofilariasis	*Dirofilaria repens*	Surgical removal	High	[56]
2015	India	64	F	Face	Asymptomatic swelling on the left side of the face for six months	Dirofilariasis	n.a.	Surgical removal	Acceptable	[57]
2015	Sri Lanka	21	M	Cheek	Asymptomatic nodular swelling of the left cheek for several months	Dirofilariasis	*Dirofilaria repens*	Surgical removal	High	[58]
2015	Sri Lanka	57	F	Cheek	Eight-month-old, slightly sensitive, firm lump in the left cheek	Dirofilariasis	*Dirofilaria repens*	Surgical removal	High	[58]
2016	Texas (USA)	79	M	Cheek	Slowly growing infiltrative mass in the right buccal space	Dirofilariasis	n.a.	Surgical removal	Acceptable	[59]
2018	Bulgaria	37	M	Cheek	Swelling of the left buccal mucosa which spontaneously changes its size for four months	Dirofilariasis	*Dirofilaria repens*	Surgical removal	High	[60]
2018	India	37	F	Cheek	Swelling in the right cheek for two months	Dirofilariasis	*Dirofilaria repens*	Surgical removal	High	[61]
2018	Italy	51	F	Cheek	Six-month swelling of the left cheek due to a noninflammatory submucosal nodule in the left maxillary vestibule	Dirofilariasis	n.a.	Surgical removal	Acceptable	[62]
2019	India	26	M	Jaw	Recurrent painful swelling of the right mandible for six months	Dirofilariasis	n.a.	Surgical removal and prescription of diethylcarbamazine and albendazole	Acceptable	[63]
2019	Serbia	45	M	Cheek	Striking edema of buccal mucosa which caused an asymmetrical deformity of the face	Dirofilariasis	*Dirofilaria repens*	Surgical removal	High	[64]
2020	France	46	F	Cheek	One-week creeping dermatitis on the eyelid followed by a submucosal nodule in the cheek	Dirofilariasis	*Dirofilaria repens*	Surgical removal	High	[65]
2022	Croatia	41	M	Lip	Asymptomatic swelling of the lower lip for more than two months	Dirofilariasis	*Dirofilaria repens*	Surgical removal	High	[66]
2022	India	50	F	Cheek	Painless nodular swelling on the left cheek for one month	Dirofilariasis	n.a.	Surgical removal	Acceptable	[67]
2022	India	26	F	Cheek	Swelling of one-year duration on the right cheek with multiple episodes of itching and graded fever especially during the night	Dirofilariasis	n.a.	Surgical removal	Acceptable	[68]
2022	India	5	M	Cheek	Slow-growing swelling of two months on the right side of the face	Dirofilariasis	n.a.	Surgical removal	Acceptable	[69]
2001	China	46	M	Jaw	Diffuse, insensitive submucosal swelling on the right upper jaw	Filariasis	n.a.	Surgical removal	Acceptable	[70]
2017	India	44	F	Cheek	Pain for six months in mandible and maxilla	Filariasis	n.a.	Surgical removal and prescription of diethylcarbamazine	Acceptable	[71]
2001	India	34	F	Cheek	Asymptomatic swelling of the right buccal mucosa	Lymphatic filariasis	*Wuchereria bancrofti*	Surgical removal and prescription of diethylcarbamazine	High	[72]
2007	India	35	F	Gingiva	Rapidly increasing swelling of the mandible and face for a month	Lymphatic filariasis	*Wuchereria bancrofti*	Fine needle biopsy	High	[73]
2010	India	50	F	Cheek	Two weeks of submucosal, mobile, firm lump in the left cheek	Lymphatic filariasis	*Wuchereria bancrofti*	Surgical removal	High	[74]
1986	Nigeria	13	F	Cheek	Painless nodule in the left cheek	Onchocerciasis	*Onchocerca volvulus*	Surgical removal	High	[75]
1985	Tanzania	62	M	Floor of the mouth	Hardening and ulcerating lesion of the floor of the mouth	Squamous cell carcinoma and lymphatic filariasis	*Wuchereria bancrofti*	n.a.	Acceptable	[76]
1985	Tanzania	54	M	Lip	Non healing ulcer in the upper lip	Squamous cell carcinoma and lymphatic filariasis	*Wuchereria bancrofti*	n.a.	Acceptable	[76]
1998	India	45	M	Cheek	Painful, rapidly growing swelling on the right maxilla for three months	Squamous cell carcinoma and lymphatic filariasis	*Wuchereria bancrofti*	Fine needle biopsy	High	[77]

N.B.: M = male; F = female

Dirofilariasis was most commonly reported from the WHO European Region (53.9%; 55/102), and the WHO South-East Asian Region (37.3%; 38/102) followed by a few reports from the WHO Region of the Americas (4.9%; 5/102), the WHO Eastern Mediterranean Region (2.9%; 3/102) and the WHO Western Pacific Region (1%; 1/102) (Table 1). The median age of dirofilariasis cases was 39 years (IQR: 28 to 52 years) and females and males were equally often affected (49% [49/101] and 51% [52/101], respectively). Oro-facial dirofilariasis was almost exclusively caused by *D*. *repens* (98%; 81/83) and only two cases were caused by *D*. *tenuis* (2%); however, it is of mention that definitive *Dirofilaria* species diagnosis was not performed in 18.6% of cases (19/102). The most commonly affected oro-facial site was the cheek (63.7%; 65/102), followed by the face (17.7%; 18/102), the lip (7.8%; 8/102) and the jaw (6.9%; 7/102); furthermore, there was each a case with involvement of the nasolabial region, the oral cavity, the soft palate and the tongue, respectively. Although in 37% (38/102) of articles there was no clear description of the main pathology of oro-facial dirofilariasis, among the remaining 64 articles asymptomatic manifestations constituted almost 80% (50/64) and only about 20% (14/64) of articles described a symptomatic case. Among asymptomatic manifestations 58% (29/50) were nodules and 42% (21/50) were swellings. On the contrary, among the symptomatic manifestations there were more swellings than nodules (71% [10/14] and 29% [4/14], respectively) and pain was the main reported symptom in 64.3% (9/14) in symptomatic oro-facial dirofilariasis cases, followed by pruritus (28.6%; 4/14) and dysesthesia (7.1%; 1/14). Therapy was not reported in almost half of oro-facial dirofilariasis cases (46%; 47/102). Cases for whom therapy was reported (n = 55) mostly underwent surgical removal (89%; 49/55) or surgical removal including anti-filarial treatment (5%; 3/55). In two cases the swelling was squeezed, and a worm was recovered and lastly in one case the nodule developed into an abscess, which burst and revealed a white worm. Further details are denoted in Table 2.

Characteristics of the nine (n = 9) oro-facial non-dirofilariasis cases are listed in Table 1. All of them were reported from tropical or subtropical regions with active disease transmission: three out of three (100%) cases of parasitologically confirmed lymphatic oro-facial filariasis and one out of three (33%) cancer cases with parasitologically confirmed concomitant lymphatic oro-facial filariasis stem from the South-East Asian Region of the WHO; one out of one (100%) case of oro-facial onchocerciasis and two out of three (67%) cancer cases with parasitologically confirmed concomitant lymphatic oro-facial filariasis stem from the WHO African region. The age ranged from 13 years to 62 years and there were 5 males (56% and 4 females (44%). All cases of oro-facial lymphatic filariasis (with or without concomitant cancer) were caused by *W*. *bancrofti*. More than half of them caused asymptomatic manifestations (56%; 5/9). Among the symptomatic cases (n = 4) pain and ulcer were most common (50%; 2/4 and 50%; 2/4, respectively). The cheeks were the most frequently reported site of disease manifestation (56%; 5/9) followed by the jaw (11%; 1/9), the lip (11%; 1/9), the gingiva (11%; 1/9) and the floor of the mouth (11%; 1/9). All oro-facial, non-dirofilariasis cases had the worm removed by a medical procedure. Further details are denoted in Table 2.

The methodological quality of articles was favorable for two thirds of the included article population (66%; 73/111) and unfavorable (i.e. low) for a third (33%; 38/111). Among the articles rated favorable, the majority was rated as having high methodological quality (64%; 47/73) and 36% (26/73) had acceptable quality.

When looking at absolute numbers and relative percentages of oro-facial filariasis cases by time and WHO region it is apparent that between 1864 and 1989 most cases were reported by the WHO European Region (83.3%; 20/24), followed by the WHO African Region (12.5%; 3/24) and lastly, the WHO South-East Asian Region (4.2%; 1/24) (Table 3). Within the period of 1990 to 1999, the WHO European Region still reported about two thirds of the total global disease burden (63%; 17/27), followed by the WHO South-East Asian Region (29.6%; 8/27) and lastly, by the WHO Region of the Americas (7.4%; 2/27). During the time frame between 2000 and 2009, each 40% of cases were reported by the WHO European Region (10/25) and WHO South-East Asian Region (10/25), each 8% of cases were reported by the WHO Western Pacific Region (2/25) and the WHO Eastern Mediterranean Region (2/25), followed by 4% (1/25) in the WHO Region of the Americas. In the most recent time frame between 2010 until present, approximately two thirds were reported by the WHO South-East Asian Region (68.6%; 24/35), followed by the WHO European Region (22.9%; 8/35), the WHO Region of the Americas (5.7%; 2/35) and lastly, the WHO Eastern Mediterranean Region (2.9%; 1/35).

**Table 3:** Number and relative percentage of oro-facial cases of filariasis per country and year.

WHO region	Country	Number and relative percentage of oro-facial cases of filariasis							
		1864 to 1989		1990 to 1999		2000 to 2009		2010 to present	
		n	%	n	%	n	%	n	%
European Region	England	0	0.0%	1	3.7%	0	0.0%	1	2.9%
	Russia	3	12.5%	3	11.1%	4	16.0%	1	2.9%
	Bulgaria	0	0.0%	0	0.0%	0	0.0%	1	2.9%
	Croatia	0	0.0%	0	0.0%	0	0.0%	1	2.9%
	Serbia	0	0.0%	0	0.0%	0	0.0%	1	2.9%
	France	5	20.8%	4	14.8%	1	4.0%	2	5.7%
	Italy	10	41.7%	8	29.6%	0	0.0%	1	2.9%
	Austria	0	0.0%	0	0.0%	3	12.0%	0	0.0%
	Greece	1	4.2%	1	3.7%	1	4.0%	0	0.0%
	Turkey	0	0.0%	0	0.0%	1	4.0%	0	0.0%
	Georgia	1	4.2%	0	0.0%	0	0.0%	0	0.0%
South-East Asian Region	Sri Lanka	1	4.2%	6	22.2%	7	28.0%	2	5.7%
	India	0	0.0%	2	7.4%	3	12.0%	22	62.9%
Western Pacific Region	China	0	0.0%	0	0.0%	2	8.0%	0	0.0%
Eastern Mediterranean Region	Tunisia	0	0.0%	0	0.0%	0	0.0%	1	2.9%
	Iran	0	0.0%	0	0.0%	2	8.0%	0	0.0%
Region of the Americas	Florida (USA)	0	0.0%	2	7.4%	1	4.0%	0	0.0%
	Texas (USA)	0	0.0%	0	0.0%	0	0.0%	1	2.9%
	Brazil	0	0.0%	0	0.0%	0	0.0%	1	2.9%
African Region	Tanzania	2	8.3%	0	0.0%	0	0.0%	0	0.0%
	Nigeria	1	4.2%	0	0.0%	0	0.0%	0	0.0%

## Discussion

The impact of filariasis on oral health is still largely unknown and epidemiological research both on filarial diseases and oral health are neglected. This comprehensive systematic review corroborates this assumption. While the earliest reported cases stem from as early as 1864 since then, only case reports, case series or review articles about these cases were published, but not a single epidemiological or large clinical study. Similarly, not a single, registered study on oro-facial filariasis was identified on large clinical trial platforms. This indicates that the topic of filarial oro-facial disease is indeed a neglected research topic and that the true rate of people suffering from oro-facial filarial disease might be immensely underestimated by the published literature. Our systematic review on the published literature suggests that the vast majority of oro-facial filariasis is represented by oro-facial dirofilariasis caused by *D*. *repens*. This is followed by a small non-dirofilarial minority represented by *W*. *bancrofti* and *O*. *volvulus*.

Oro-facial dirofilariasis manifested exclusively as nodules or swellings in different tissue locations. This is in line with the biology of the adult stages of *D*. *repens* and *D*. *tenuis*, both of which are known to be most-commonly located in subcutaneous tissue [2]. While swellings (some of which can appear to migrate) are often described to be caused by the active migration of the adult worm, subcutaneous nodules are formed by adult worms whose migration is rendered stationary by the host’s immune system [17,78]. Characteristically, many case reports reported swellings of subcutaneous tissue for weeks to months, which often spontaneously increased and decreased in size and finally manifested into a single, mobile, soft, or firm nodule. Also, in line with the literature these nodules and swellings are mostly asymptomatic, however, can become painful if the immune system of the host attempts to clear the active infection [79]; again, this is congruent with our findings since pain and dysesthesia combined constituted 71% (10/14) of symptoms in symptomatic oro-facial dirofilariasis. Interestingly, most oro-facial dirofilariasis manifestations occurred in the cheek and face and to lesser frequency in the lip, jaw and elsewhere. However, it is believed that these are not true predilection sites, but artefacts resulting from categorizing the human body into anatomical regions, some of which are naturally larger than others. From this perspective the stochastic probability that a migrating worm is found in the larger tissue regions of the cheek or face is higher than in the comparatively smaller tissue regions of the jaw, or lip. Furthermore, virtually all cases had the worm removed, either iatrogenically (98%; 54/55) or spontaneously (2%; 1/55) which is again in line with the recommended treatment of general dirofilariasis caused by *D*. *repens* or *D*. *tenuis* which is surgical removal [79].

The vast majority of dirofilariasis was caused by *D*. *repens* (98%), followed by *D*. *tenuis* while no case was caused by *D*. *immitis*. This phenomenon can be explained via parasite biology since adults of *D*. *immitis* are described to inhabit the intravascular space primarily of pulmonary arteries, and not subcutaneous tissue (which is the case for adults of *D*. *repens* and *D*. *tenuis*) [2].

Interestingly, while *D*. *repens* is described to exclusively occur in the old world, there was one case of oro-facial dirofilariasis reported from the Americas caused by *D*. *repens*. The authors report that even despite careful questioning, the patient did not recall any insect bite, nor did she report any history of recent travel outside of the United States [33].

Similarly to *D*. *repens* and *D*. *tenuis*, also *L*. *loa*, *O*. *volvulus* and certain *Mansonella spp*. actively migrate through tissue. However, except for one detected onchocerciasis case we did not detect any case caused by the other above-mentioned filarial species. Although we did neither detect a single case of tooth pain nor a case of oro-facial loiasis, we recently submitted a secondary analysis of a large cross-sectional survey for publication indicating that *L*. *loa* can cause transient tooth pain [5]; we argued that a transient swelling of the periodontium or the soft tissue of the oral cavity may explain this symptom. While this phenomenon needs to be corroborated by further research it suggests that up to 25% of individuals with loiasis may suffer from transient tooth pain [80].

On the other hand, filarial pathogens, such as *W*. *bancrofti*, *B*. *malayi and B*. *timori* can block lymphatic vessels, thereby potentially exerting secondary damage onto other organs, for example, by causing severe lymphedema of the limbs, which is often accompanied by diffuse thickening of the skin and the subcutaneous tissue; this is then called elephantiasis. We did not detect any case of elephantiasis of filarial origin in the oro-facial region. Yet, we found one publication which described a case of filarial infection with lymphedema in the lip. However, we did not include this article in our analysis population, because the oro-facial pathology was highly likely caused by bacteria and not by the filarial infection [81].

Oro-facial filarial disease was more common in adults than in children. This is epidemiologically and biologically plausible due to two factors: first, the sometimes-long exposition period required to acquire active infection and second, the generally long induction time required for filarial infections to manifest in filarial disease [1,82,83].

Time trend analyses seem to indicate that the share of global oro-facial filariasis burden carried by the WHO European Region was about 80% until 1989 and gradually declined thereafter to approximately 20%. On the contrary, the share of global disease burden of the WHO South-East Asian Region was below 5% until 1989 and eventually increased to almost 70%. Other WHO regions consistently had comparatively low shares of global case burden over time. While the relative increase from South-East-Asian countries seems plausible, due to the co-endemicity of various filarial pathogens there, it seems puzzling that other regions, especially the WHO African Region reported such consistently low rates of oro-facial filariasis given that sub-Saharan African countries carry a comparatively rather high overall filarial case burden [84,85]. The most likely explanations for this phenomenon also represent the main limitations of this article. First, reported case numbers need to be interpreted with caution, since case counts do not stem from routine, standardized and objective surveillance systems but taken from the published literature. This means that the probability of case reporting depends much more on the interest and industriousness of a physician, or researcher than on the actual presence of oro-facial filariasis in a given setting. In other words, the subjective nature of case reporting does not only limit direct comparability of case burden among countries, but also across time. This highlights the importance of conducting large and objective epidemiological studies which should consider and ascertain all locally endemic filarial infections when assessing their potential impact on oro-facial health and the overall public health importance of oro-facial filariasis. Second, the scope of this review encompassed the whole world. Given the high global heterogeneity of the quality of health care systems in different countries this even further reduces comparability of case burden among countries, because it is more likely that articles get published in high-resource settings than in low-resource settings, owing to a relatively better research infrastructure.

It is of mention that in the majority of reported cases asymptomatic swellings, or nodules were the main symptom of oro-facial filarial disease. An important differential diagnosis for asymptomatic swellings, or nodules is cancer. In fact, three cases even had cancer and oro-facial filariasis at the same time. Unfortunately, due to a lack of published studies it is not clear whether oro-facial filariasis might lead to oro-facial cancer, or whether (even though it seems unlikely) cancer might pre-dispose to acquisition of filarial infection, or whether this was merely due to chance. Further prospective studies need to be conducted to investigate any potential causal relationship. However, the similarity of symptomatology indicates that cases of oro-facial filariasis should receive appropriate assessment for cancer and vice versa. Yet, most cases of overall filarial disease are almost exclusively reported from lower- and middle-income countries from the tropics, where health systems often suffer from a lack of resources. Thus, it might be problematic for the global majority of patients with oro-facial filariasis to receive adequate cancer care. Therefore, in the absence of adequate diagnostic facilities and diagnostic apparatus simple cross-sectional studies could be conducted in resource-limited settings to provide oral health providers with valuable information to be ultimately used for case management. First, the prevalence of oro-facial lesions, as well as, their causes should be investigated. Second, symptom scores could be computed which could be used to create guidelines for the medical expert to manage the given oro-facial health problem in a specific setting. Meanwhile, in the absence of adequate clinical and epidemiological research results, oral health providers should consider parasitological etiologies in oro-facial disease manifestations particularly in patients with high risk of carrying a specific parasitic infection. Unfortunately, oral health providers were often not exposed in dental medicine curricula to the idea that parasites can be an underlying cause of certain oro-facial diseases. This educational gap is even further complicated by the fact that parasite-specific characteristics (e.g. life cycles, risk factors, or disease manifestation) can be vastly different among parasitic diseases. Therefore, closing this gap would probably demand a disproportionate exposure of dental medicine students to parasitology at the expense of other relevant educational content in the syllabus. Whether this is justified needs to be judged by (dental) medical faculties depending on the geographical area of the respective university considering local parasitic disease transmission rates.

## Conclusion

Although filarial diseases are to date not generally regarded as being associated with oral health problems this assumption might not be justified. This comprehensive systematic review was conducted to detect and collate all published studies on oro-facial filariasis. The fact that only case reports, or case series were identified, while not a single, large epidemiological or clinical study was detected suggests that this constitutes a neglected field of research. Cases identified in the published literature indicate that the vast majority of published oro-facial, filarial case reports were cases of dirofilariasis. However, it might well be that the phenomenon of oro-facial filariasis presents completely different in the context of large epidemiological and clinical studies if all locally endemic filarial infections are considered and ascertained in an objective and systematic manner. Therefore, such large studies are important and warranted to understand oro-facial filariasis better as well as its potential public health importance. Among the published studies, oro-facial filariasis manifested exclusively as nodules or swellings in different tissue locations. These nodules and swellings were mostly asymptomatic and therefore, cancer is an important differential diagnosis.
